# Response of the consumers to the menu calorie-labeling on online food ordering applications in Saudi Arabia

**DOI:** 10.1186/s40795-024-00829-x

**Published:** 2024-02-02

**Authors:** Sarah Alkhunein, Wejdan Alghafari, Haya Alzeer, Omar Alhumaidan, Sarah Alsalman, Nojoud Alshathry, Areej Alkhaldy

**Affiliations:** 1 National Nutrition Committee (NNC), Saudi Food and Drug Authority (SFDA), Riyadh, Kingdom of Saudi Arabia; 2 Department of Clinical Nutrition, Faculty of Applied Medical Sciences, King Abdulaziz University, Jeddah, Kingdom of Saudi Arabia; 3 Department of Community Health Sciences, College of Applied Medical Sciences, King Saud University, Riyadh, Kingdom of Saudi Arabia

**Keywords:** Calories, Labeling, Applications, Delivery, Menu

## Abstract

**Background:**

The use of online food ordering applications is widespread; however, to date, there have been no studies on the effect of the menu calorie labeling in online food ordering applications on the consumers’ dietary habits and food choices in Saudi Arabia. Therefore, this study aimed to evaluate the response of the consumers to the menu energy-labeling on online food ordering applications in Saudi Arabia by exploring: (a) the consumers’ preference and frequency of ordering from online food applications; (b) the consumers' self-reported knowledge, awareness, and interest towards menu calorie information on online food ordering applications; (c) the impact of menu calorie information on online food ordering applications on consumers’ food choices.

**Methods:**

This is a cross-sectional study conducted between October and November of 2022. A total of 419 participants were recruited using an online questionnaire.

**Results:**

The findings showed that most participants (59%) preferred to order using online food ordering applications. Saving time and effort are the main reasons for using online food ordering applications (61%). Nearly half of the participants (45%) were interested in viewing calorie information on online food ordering applications menus and (47%) did notice calorie information displayed on the menu when ordering from an online food ordering application. Calorie information in online food ordering applications menus is primarily utilized to monitor intake for weight maintenance (19%). The ability to calculate energy requirements and interest in viewing calorie information on online food ordering applications menus were linked to younger age and a higher level of education (p < 0.05).

**Conclusions:**

Overall, consumers showed limited awareness and interest towards the menu calorie information displayed in the online food ordering applications. These findings highlight the importance of developing public health programs to increase public awareness about calorie labeling on menus to aid in the success and effectiveness of the calorie labeling in online food ordering applications as a tool to promote balanced energy intake. Further research is needed to understand the most effective way to deliver calorie information to consumers on an online food ordering application.

**Supplementary Information:**

The online version contains supplementary material available at 10.1186/s40795-024-00829-x.

## Background

In recent years, menu calorie-labelling and obesity topics have received significant attention. Obesity is primarily caused by an imbalance between energy intake and expenditure [1]. There is growing evidence about the role of menu calorie-labelling in influencing consumers' choices which could reduce the risk of obesity by promoting healthy food choices. It has been reported that obesity is a risk factor for many diseases, including diabetes mellitus, hypertension, and cardiovascular disease [2]. The prevalence of obesity has approximately tripled globally since 1975, and in Saudi Arabia obesity prevalence in those aged 15 years and older is 24.1% among males and 33.5% among females [3, 4]. Considering its association with several health issues, it is vital to investigate the factors that affect dietary behavior [5]. Dietary behavior is a broad term that encompasses food choices, dietary intake, and eating behavior. A single term for dietary behavior does not exist, as it is shared among various disciplines such as nutrition, epidemiology, and psychology [6]. Six determinants have been identified as influencing food choices: biological (hunger, appetite, and taste), economic (cost and income), physical (access, education, skills, and time), social (class, culture, and social context), psychological (mood, stress, and guilt), and attitudes, beliefs, and knowledge of food [7].

In terms of physical determinants, access to food stores and the availability of items within them affect food choices [7]. The consumption of food prepared away from home is increasing, especially among children and young adults [8]. In addition, the economic growth in recent decades has led to a cultural shift to a modern Westernized lifestyle [9]. It has become an investment target for owners or franchisers of fast-food chains, restaurants, and cafés. The food service market would expand by 6% over the next 5 years in Saudi Arabia [10]. Owing to market growth, a mediator is needed to facilitate access to various restaurants.

Online food ordering applications and third-party platforms act as intermediaries between customers and restaurants [11]. A recent study reported the reasons for using the online food ordering applications which include easier accessibility, faster services, variety of restaurant options, effects of advertisements, quality of service, individuals with long working hours, food menu photos, and the impact of social media [12].

As online food ordering applications became widespread and more people started to use them, it was important for different government agencies such as the Saudi Food and Drug Authority (SFDA) to implement a menu calorie labeling policy to inform customers and help them consider better choices. In August 2018, the SFDA enacted the “Putting Calories on Food Establishments Menu’s Selling Away-From-Home Foods” policy. It applies to all food establishments, such as restaurants, cafés, ice cream shops, juice shops, bakeries, and cafeterias. The regulation requires displaying calorie information in a clear manner beside each type of food. Some food types were excluded, such as temporary and daily foods and special orders. The menu calorie labeling policy for restaurants is a cost-free strategy that can prevent obesity and other chronic diseases [13].

Since the menu calorie labeling policy was mandated at the end of 2018, several studies have assessed its effects on food choices. A cross–sectional study investigated the impact of calorie information in online food ordering applications on the selection of meals by participants during COVID-19. They found that 60% of the participants did not consider calories, whereas only 13% paid attention to calories in the menu [14]. In addition, another study assessed the community’s perspective on displaying calories on online food ordering applications; approximately 34% of respondents revealed that they would choose low-calorie items, 26% would not choose low-calorie items, and 34% may choose low-calorie items [15]. Another study compared before and after the implementation of a menu calorie labeling policy based on the sales of a famous fast-food chain from dine-in and online orders via different platforms. The data showed a slight increase in the average total number of calories per order, approximately 1% for dine-in orders and 7% for online orders. There was a slight decrease in the average beverage calories in both settings and fries in dine-in orders. The average calories per order on online platforms were 10% higher than dine-in orders prior to the policy implementation, and 16% higher after the policy implementation. However, there was no statistically significant impact of the policy implementation on the total number of calories per order [16].

In Saudi Arabia, the number of online food ordering applications and individuals who are using them are increasing [17]; with no study examining the effect of menu calorie labeling in online food ordering applications on the consumers’ dietary habits and food choices in Saudi Arabia. Therefore, this study aimed to evaluate the response of the consumers to the menu energy-labeling on online food ordering applications in Saudi Arabia by exploring: (a) the consumers’ preference and frequency of ordering from online food applications; (b) the consumers' self-reported knowledge, awareness, and interest towards menu calorie information on online food ordering applications; (c) the impact of menu calorie information on online food ordering applications on consumers’ food choices. The results of this study could help policymakers better comprehend the response of Saudis to the menu calorie labeling policy, in order to set priorities to modify the policy in Saudi Arabia. It will also benefit local researchers who want to assess how the policy has impacted the public over time. In addition, this study is important for international researchers interested in the effect of country-specific factors on policy implementation.

## Methods

### Study design

This was a cross-sectional, an online questionnaire-based study. The study was conducted between October and November 2022. This study was approved by the Biomedical Ethics Research Committee of King Abdulaziz University (Jeddah, Saudi Arabia) (Reference No. 370 − 22). An online informed consent form was signed electronically by all participants of the study before commencing with the online survey. Password-protected files on secure servers were used to retain the participants' information, and no information was shared with a third party without the principal investigator’s written agreement.

### Participants and recruitment

A total of 419 adult nationals and residents of the Kingdom of Saudi Arabia aged 18 years or older, who have used online food ordering applications were included as a convenience sample. During data collection, participants’ actual use of these applications was verified through a question at the beginning of the questionnaire. The participants were required to respond to an online survey conducted using Google Forms. Snowball sampling was used to distribute the online surveys through several social media sites, including Twitter and WhatsApp. The National Nutrition Committee’s account tweeted the survey link, while the authors distributed the survey link via WhatsApp to all individuals on their personal contact lists, including family members and friends, and requested that they do the same for everyone they knew who lived in Saudi Arabia.

### Sample size calculation

The calculation for sample size was performed to estimate the frequency of responses based on Saudi adults numbering 25,828,206 (≥ 18 years old) as reported by the Saudi General Authority for Statistics in 2019 [18]. The online Epi Info sample size calculator (Division of Health Informatics and Surveillance, and Centre for Surveillance, Epidemiology and Laboratory Services, GA, USA) was used to determine the required sample size by assuming that 50% of target individuals would agree to participate in the study, with a confidence level of 95%, margin error of 5% and design effect of 1. Consequently, the total calculated sample size was 385 participants [19].

### Study questionnaire

The questionnaire was developed for this study by the study research team with a nutritional background to collect relevant information from the participants (see Additional file 1). The questionnaire was assessed through experts’ evaluations (n = 8) in the field of nutrition. In addition, a pilot study was conducted to empirically test the questionnaire with 10 people to ensure the clarity of the questions. The questionnaire was revised based on experts’ feedback, public feedback, and comments. Briefly, modifications made to the pilot questionnaire included changing response options and rewording, adding, and removing some questions. As a result of the reviewing process, the final questionnaire was composed of five main sections, totaling 24 questions, and took approximately ten minutes to complete.

The first section of the questionnaire included questions regarding sociodemographic characteristics of the participants including gender, age, marital status, level of education, monthly income, job status, field of study, medical status, and self-reported weight and height (used to calculate BMI). The second section inquired about the preference of ordering from online food ordering applications, the possible reasons for ordering from these applications, and to what extent the frequency of use of these applications increased during the COVID-19 pandemic.

The third section of the questionnaire gathered information about the types of restaurants that frequently utilized online food ordering applications, the main reasons for choosing a specific restaurant, the most frequently ordered meal from these applications, as well as the frequency of ordering food from online food ordering applications, and the most frequent time for ordering from these applications. In addition, the participants were asked when they ordered food from the applications, whether they would order for themselves or family members or friends.

The fourth section was about the consumers’ self-reported knowledge, awareness, and interest towards calorie information on online food ordering application menus. The questions in this section asked the participants whether they knew how to calculate their daily calorie requirements, if they were interested in viewing calorie information on online food ordering application menus, and if they noticed any calorie information displayed on the menus when ordering from these applications.

In the last section, we inquired about the impact of displaying calories in online food ordering applications on consumers’ food choices. The section included questions about whether displaying calorie information on menus in online food ordering applications could have positively affected the food choices. In addition, the reasons for using calorie information on menus in these applications were examined.

### Statistical analysis

Data were checked and entered using standardized entry codes written in the Statistical Package for the Social Sciences (SPSS, Version 23.0; IBM Corp., Armonk, NY, USA) v26 data file. Data were presented as frequencies and proportions. Descriptive statistics were used to present demographic characteristics and consumers’ preference and frequency of ordering from online food ordering applications data as number and percentage. To test the difference between two variables (selected sociodemographic variables including gender, marital status, job status, field of study, monthly income, and BMI with other variables either the knowledge of how to calculate personal daily calorie requirements, or noticing calorie information displayed on the food ordering application menus, or the public’s interest in viewing calorie information on online food ordering application menus), a chi-square test of independence was used. In addition, independent samples t-test and one-way analysis of variance test were applied to compare arithmetic mean of two or more than two different groups, respectively with a p-value < 0.05 denoting statistical significance. The Bonferroni correction was used for multiple comparisons.

## Results

### Participant characteristics

A total of 419 food consumers participated in this study. Their sociodemographic characteristics are summarized in Table 1. Most of the participants were female (83.1%), and their ages ranged between 18 and 72 years (mean = 30.2 years; standard deviation = 10.5 years). Almost two-thirds were single (61.3%), and held a bachelor’s degree (66.1%). More than one-third (37%) were employed, and 33.4% were students. 22% of the participants were in the medical field and 17.9% were in nutrition and food sciences, while the remaining participants had a range of different backgrounds (Table 1). The monthly income of 34.9% of the participants was below 2000 Saudi Riyals (533$ USD) per month, whereas 23.4% exceeded 10,000 Saudi Riyals (2666$ USD) per month. The prevalence rates of overweight and obesity among the participants were 25.3% and 16%, respectively. A history of chronic health illness was reported by 19.6% of the participants.

**Table 1:** Sociodemographic characteristics of the participants (n = 419)

Variables	N	%
**Age, y**		
Range Mean ± SD	18–72 30.2 ± 10.5	
**Gender**		
Male Female	71 348	16.9 83.1
**Marital status**		
Single Married Divorced Widowed	257 145 10 7	61.3 34.6 2.4 1.7
**Highest educational level**		
Below secondary school Secondary school/equivalent Bachelor`s degree Postgraduate degree	16 78 277 48	3.8 18.6 66.1 11.5
**Job status**		
Student Employed Unemployed Self-employed Retired	140 155 101 8 15	33.4 37.0 24.1 1.9 3.6
**Field of study**		
Nutrition/food sciences Medical Science Literacy Management Non-specific	75 92 41 42 69 100	17.9 22.0 9.8 10.0 16.5 23.8
**Monthly income, SR (US dollars)**		
< 2000 (< 533$ USD) 2000–5000 (533–1333$ USD) 5001–7000 (1334–1866$ USD) 7001–10,000 (1867–2666$ USD)	146 104 44 27	34.9 24.8 10.5 6.4
> 10,000 (> 2666$ USD)	98	23.4
**Body Mass index** ^a^		
Underweight Normal Overweight Obesity	31 215 106 67	7.4 51.3 25.3 16.0
**Reported living with a chronic illness**		
No Yes	337 82	80.4 19.6

n (%) = Data presented as number and percentage; SR = Saudi Riyal

^a^Self-reported weight and height were used to calculate BMI. The BMI categories are defined as follows: healthy weight (BMI 18.5 to 24.9 kg/m2), overweight (BMI 25.0 to 29.9 kg/m2), and obese (BMI ≥ 30 kg/m2)

### Preference of ordering from online food ordering applications

More than half of the participants (58.9%) preferred ordering from online food ordering applications rather than directly from restaurants. The reported reasons for this included saving time and effort (61.3%) and ease of access to restaurants and shops compared to direct ordering from a restaurant (53.4%). Most participants (64%) claimed that their use of online food ordering applications increased during the COVID-19 pandemic (Table 2).

**Table 2:** Preference of ordering from online food ordering applications (n = 419)

	n	%
**1. Do you prefer to order directly from the restaurant or online food ordering applications? (n = 419)**		
Online food ordering applications Restaurants	247 172	58.9 41.1
2. What is the reason behind using online food ordering applications? (n = 247)*		
The different price and offers compared with ordering directly from the restaurant Saving time and effort The ease of access to restaurants and shops compared with ordering from a restaurant directly The variety of options for restaurants and shops available on the apps	99 257 224 172	23.6 61.3 53.4 41.1
3. **Has your use of online food ordering applications increased during the COVID-19 pandemic? (n = 419)**		
No Yes	151 268	36.0 64.0

n (%) = Data presented as number and percentage

*Participants were allowed to choose more than one option

### Frequency of ordering from online food ordering applications

The restaurants most frequently utilized via online food ordering applications were those serving fast food (69.7%), followed by coffee shops (47%) and those serving desserts (40.8%), traditional food (39.9%), and international food (39.6%). The main reasons for choosing a specific restaurant were taste (61.6%), craving for a specific food (59.4%), meal price (45.6%), delivery price (43.4%), and level of hygiene and quality (43.2%). The most frequently ordered meal from online food ordering applications was dinner (65.8%). More than half (53%) of the participants used online food ordering applications once per week, and the most frequent time for ordering from online food ordering applications was in the evening (7 pm– 12 am) (61.6%). Most participants (78.1%) ordered food for themselves and family members or friends (Table 3).

**Table 3:** Types of restaurants in online food ordering applications and frequency of ordering by the participants (n = 419)

	n	%
1. What type of restaurants or shops do you order from?*		
Fast-food restaurants International food restaurants Traditional food restaurants Healthy food restaurants Vegetarian restaurants Desserts shops Beverages shops Coffee shops Others	292 166 167 97 12 171 100 197 25	69.7 39.6 39.9 23.2 2.9 40.8 23.9 47.0 6.0
2. What is the reason behind choosing a specific restaurant from the application?*		
Meal price Delivery price Delivery speed Healthiness Taste Reviews from others Pictures displayed on menus Craving a specific food The desire of others to eat certain food Level of hygiene and quality Other reasons	191 182 125 70 258 101 68 249 159 181 20	45.6 43.4 29.8 16.7 61.6 24.0 16.2 59.4 37.9 43.2 4.8
3. **What type of meal do you order the most from online food ordering applications?**		
Breakfast Lunch Dinner Snacks Beverages	5 85 276 41 12	1.2 20.3 65.8 9.8 2.9
4. **How frequently do you use online food ordering applications?**		
Once per week 2–4 times per week 5–6 times per week Once per day 2 or more times per day	222 167 16 11 3	53.0 39.9 3.8 2.6 0.7
5. **What time of the day do you mostly order from online food ordering applications?**		
Morning (6 am − 12 pm) Afternoon (1 pm − 6 pm) Evening (7 pm − 12 am) Post-midnight (1 am − 5 am) No specific time	6 83 258 21 51	1.4 19.8 61.6 5.0 12.2
6. **When you order from food delivery apps, you order**:		
For myself For myself, and a family member or friend For a family member or friend only	68 327 24	16.2 78.1 5.7

n (%) = Data presented as number and percentage

*Participants were allowed to choose more than one option

### Frequency of ordering meals from online food ordering applications according to meal categories

**Fig. 1:**
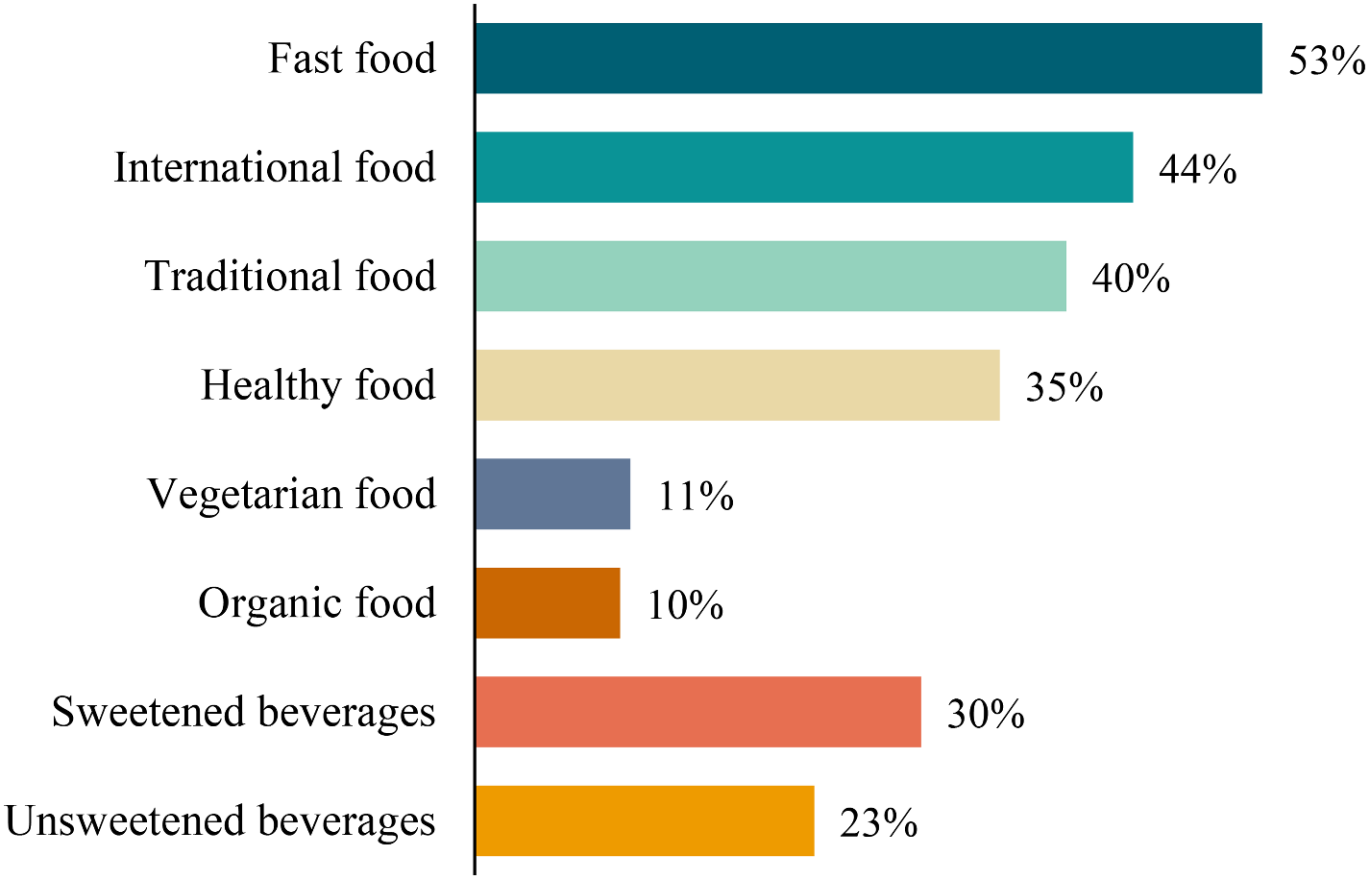
Frequency of ordering meals from online food ordering applications according to meal categories per week (n = 419)

The meals most frequently ordered from online food ordering applications per week were fast food (53.1%), international food (44.4%), traditional food (47.0%), and healthy food (35.4%) (Fig. 1).

### Participants’ self-reported knowledge, awareness, and interest towards menu calorie information on online food ordering application

Overall, slightly more than half (50.6%) of participants self-reported that they knew how to calculate their daily calorie requirements. Under half (44.9%) were interested in viewing calorie information on online food ordering application menus and 46.6% noticed calorie information displayed on the menu when ordering from an online food ordering application (Table 4).

**Table 4:** Participants’ self-reported knowledge, awareness, and interest towards menu calorie information on online food ordering application (n = 419)

	Knowledge of how to calculate personal daily calorie requirements		Noticing any calorie information displayed on the menu when ordering from an online food ordering application			Interested in viewing calorie information on online food ordering application menus		
	No	Yes	No	Sometimes	Yes	No	Sometimes	Yes
**Total**	207	212	91	195	133	102	188	129
**Gender**								
Male (n = 71) Female (n = 348)	40 (56.3) 167 (48.0)	31 (43.7) 181 (52.0)	14 (19.7) 77 (22.1)	24 (33.8) 171 (49.1)	33 (46.5) 100 (28.7)	20 (28.2) 82 (23.6)	32 (45.1) 156 (44.8)	19 (26.8) 110 (31.6)
**p-value**	0.200*		0.011*				0.617*	
**Age in years**								
Mean ± SD	31.77 ± 11.83	28.63 ± 8.73	32.70 ± 12.62	27.94 ± 8.68	31.74 ± 10.70	31.92 ± 11.77	28.82 ± 8.87	30.78 ± 11.36
**p-value**	0.002**		< 0.001***			0.040***		
**Marital status**								
Single (n = 257) Married (n = 145) Divorced (n = 10) Widowed (n = 7)	115 (44.7) 81 (55.9) 6 (60.0) 5 (71.4)	142 (55.3) 64 (44.1) 4 (40.0) 2 (28.6)	47 (18.3) 39 (26.9) 3 (30.0) 2 (28.6)	133 (51.7) 56 (38.6) 5 (50.0) 1 (14.3)	77 (30.0) 50 (34.5) 2 (20.0) 4 (57.1)	58 (22.6) 40 (27.6) 2 (20.0) 2 (28.6)	124 (48.2) 59 (40.7) 5 (50.0) 0 (0.0)	75 (29.2) 46 (31.7) 3 (30.0) 5 (41.7)
**p-value**	0.091*		0.084*			0.146*		
**Job status**								
Student (n = 140) Employed (n = 155) Unemployed (n = 101) Self-employed (n = 8) Retired (n = 15)	13 (81.2) 55 (70.5) 124 (44.8) 15 (31.3)	147 (51.6) 5 (41.7) 7 (38.9) 42 (50.0) 11 (55.0)	26 (18.6) 36 (23.2) 23 (22.8) 0 (0.0) 6 (40.0)	73 (52.1) 67 (43.2) 45 (44.6) 7 (87.5) 3 (20.0)	41 (29.3) 52 (33.5) 33 (32.7) 1 (12.5) 6 (40.0)	26 (18.6) 42 (27.1) 25 (24.8) 3 (37.5) 6 (40.0)	61 (43.6) 82 (52.9) 42 (41.6) 1 (12.5) 2 (13.3)	53 (37.9) 31 (20.0) 34 (33.7) 4 (50.0) 7 (46.7)
**p-value**	< 0.001*		0.107*			0.004*		
**Field of study**								
Nutrition/food sciences (n = 75) Medical (n = 92) Science (n = 41) Literacy (n = 42) Management (n = 69) Non-specific (n = 32) Others (n = 68)	8 (10.7) 31 (33.7) 24 (58.5) 27 (64.3) 42 (60.9) 27 (84.4) 48 (70.6)	67 (89.3) 61 (66.3) 17 (41.5) 15 (35.7) 27 (39.1) 5 (15.6) 20 (29.4)	14 (18.7) 16 (17.4) 8 (19.5) 14 (33.3) 13 (18.8) 9 (28.1) 17 (25.0)	41 (54.6) 43 (46.7) 19 (46.3) 17 (40.5) 35 (50.7) 13 (40.6) 27 (39.7)	20 (26.7) 33 (35.9) 14 (34.1) 11 (26.2) 21 (30.4) 10 (31.3) 24 (35.3)	17 (22.7) 17 (18.5) 5 (12.2) 12 (28.6) 19 (27.5) 8 (25.0) 24 (35.2)	42 (56.0) 48 (52.2) 22 (53.7) 18 (42.8) 28 (40.6) 8 (25.0) 22 (32.4)	16 (21.3) 27 (29.3) 14 (34.1) 12 (28.6) 22 (31.9) 16 (50.0) 22 (33.4)
**p-value**	< 0.001*		0.660*			0.023*		
**Monthly income, SR (US dollars)**								
< 2000 (< 533$ USD) (n = 146) 2000–5000(533–1333$ USD) (n = 104) 5001–7000(1334–1866$ USD) (n = 44) 7001–10,000(1867–2666$ USD) (n = 27) > 10,000 (> 2666$ USD) (n = 98)	78 (53.4) 48 (46.2) 20 (45.5) 18 (66.7) 43 (43.9)	68 (46.6) 56 (53.8) 24 (54.5) 9 (33.3) 55 (56.1)	33 (22.6) 19 (18.3) 10 (22.7) 5 (18.5) 24 (24.5)	72 (49.3) 55 (52.9) 16 (36.4) 13 (48.2) 39 (39.8)	41 (28.1) 30 (28.8) 18 (40.9) 9 (33.3) 35 (35.7)	38 (26.0) 24 (23.1) 10 (22.7) 10 (37.0) 20 (20.4)	59 (40.4) 48 (46.2) 17 (38.6) 10 (37.0) 54 (55.1)	49 (33.6) 32 (30.8) 17 (38.6) 7 (25.9) 24 (24.5)
**p-value**	0.194*		0.561*			0.331*		
**Body mass index**								
Underweight (n = 31) Normal (n = 215) Overweight (n = 106) Obesity (n = 67)	15 (48.4) 95 (44.2) 51 (48.1) 46 (68.7)	16 (51.6) 120 (55.8) 55 (51.9) 21 (31.3)	6 (19.4) 42 (19.5) 27 (25.5) 16 (23.9)	17 (54.8) 103 (47.9) 48 (45.3) 27 (40.3)	8 (25.8) 70 (32.6) 31 (29.2) 24 (35.8)	6 (19.4) 52 (24.2) 24 (22.6) 20 (29.9)	12 (38.7) 100 (46.5) 46 (43.4) 30 (44.7)	13 (41.9) 63 (29.3) 36 (34.0) 17 (25.4)
**p-value**	0.006*		0.747*			0.661*		

*Chi-square test **Independent samples t-test ***One-way analysis of variance test

Participants self-reported that they knew how to calculate personal daily calorie requirements were, on average, significantly younger (28.63 years) than those that did not know how to do so (31.77 years) (p = 0.002). Older participants also tended not to notice calorie information displayed on menus when ordering from online food ordering applications (32.7 years) compared with those who sometimes noticed (27.94 years) or always noticed this information (31.74 years) (p < 0.001). As shown in Table 4, participants who were not interested in viewing calorie information on online food ordering application menus were significantly older than those who were sometimes or always interested (31.92 vs. 28.82 and 30.78 years, respectively; p = 0.040).

Participants with no history of chronic health illness reported that they were more likely to know how to calculate personal daily calorie requirements than their counterparts (53.7% vs. 37.8%, p = 0.01). Participants with a normal body mass index were more likely to know how to calculate personal daily calorie requirements than were obese participants (55.8% vs. 31.3%; p = 0.006) (Table 4). Males were more likely than females to notice any calorie information displayed on the menu when ordering from online food ordering applications (46.5% vs. 28.7%; p = 0.001).

Highly educated participants were more likely to know how to calculate their personal daily calorie requirements than those with lower levels of education (p < 0.001). Those who studied nutrition or food sciences had the greatest knowledge about calculating daily calorie requirements (89.3%), whereas the lowest knowledge was observed among those who had undertaken non-specific studies (15.6%) (p < 0.001). Most highly educated participants (70.8%) expressed interest in viewing calorie information on online food ordering application menus, whereas only 42.3% of secondary school/equivalence-educated persons expressed interest in viewing such information (p = 0.001).

Half of the participants who undertook a non-specific field of study, compared to 21.3% and 29.3% of those who studied nutrition/food science and medical sciences, respectively, were interested in viewing calorie information on online food ordering application menus (p = 0.023). Half of the self-employed participants, compared with only 20% of the employed persons, were interested in viewing calorie information on online food ordering application menus (p = 0.004).

### Impact of displaying calorie information of meals on online food ordering applications on participants’ food choices

**Fig. 2:**
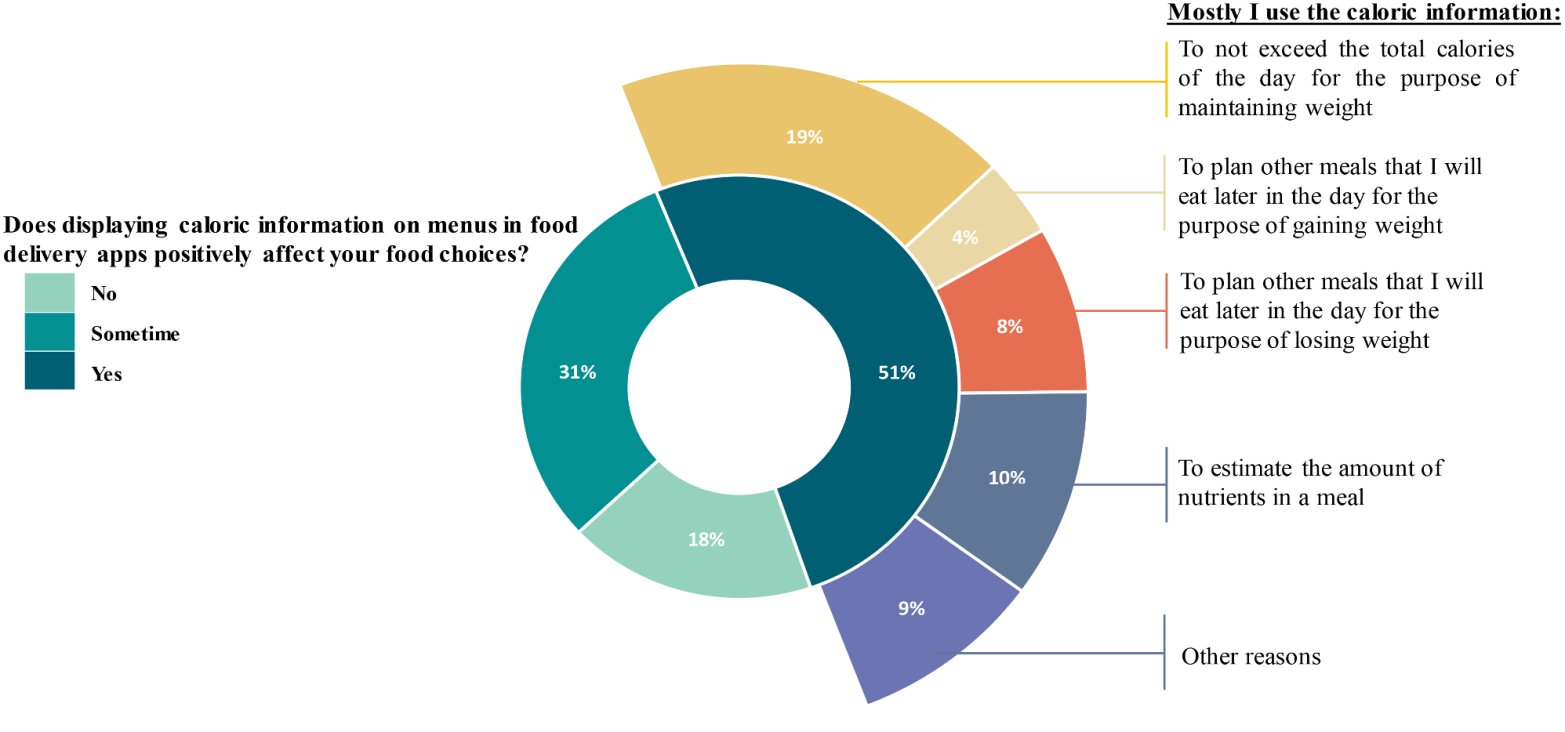
The impact of displaying calorie information of meals on online food ordering applications on consumers’ food choices (n = 328)

Displaying calorie information on online food ordering applications menus could have positively affected the food choices of 50.3% of participants (Fig. 2). More than one-third of the participants (37.5%) may have used calorie information mostly to avoid exceeding their total daily calorie allowance to maintain weight and to estimate the amount of nutrients in a meal (20.4%). Participants with postgraduate degrees were more likely than the lowest-educated participants to declare that displaying calorie information on online food ordering application menus could have positively affected their food choices (p = 0.030). Most employed participants (63%), compared with only 25% of self-employed participants, stated that displaying calorie information on online food ordering applications menus may have positively affected their food choices (p = 0.033).

## Discussion

This study is the first in Saudi Arabia to focus on the menu calorie labeling policy in online food ordering applications, and it aimed to explore the public preference and experience of ordering from online food ordering applications, their response to display the calorie information of meals on online food ordering applications menus, and whether it impacts their food choices. There were 419 questionnaire respondents with a mean age of 30 years. Almost half of them were within a normal weight range and held a university degree or higher. Overall, the result of this study showed that saving time and effort were the major reasons for using online food ordering applications.

Calorie information in online food ordering applications menus was primarily utilized to monitor intake for weight maintenance. Participants who self-reported that they knew how to calculate personal daily calorie requirements were more likely to be younger and have a higher level of education, in parallel with the characteristics of those who were interested in viewing calorie information on online food ordering applications. Online food ordering applications satisfy various social and individual customer demands, as they offer convenience and ease of use [20–23]. Accordingly, more than half of the participants (58.9%) in this study preferred to order through online food ordering applications and not from restaurants, either to save time and effort or because of the ease of access to restaurants. The result in this study is consistent with findings from other studies and a qualitative study on frequent online food ordering application users in the United Kingdom [15, 24, 25]. However, two studies in Nepal and Malaysia found that saving time, but not effort, was significantly associated with online food ordering application use [26, 27]. Food delivery services have grown rapidly as a result of the COVID-19 pandemic [28, 29]. Most participants (64%) claimed that their use of online food ordering applications increased during the COVID-19 pandemic, which is consistent with the findings of similar studies in Saudi Arabia [14, 15] but one study found no change in use [30].

Research has shown that individuals with different food-choice motives exhibit different food product preferences [31]. The primary reasons behind the respondents’ restaurant choices were taste and cravings, followed by meal and delivery prices, which are predictable but also worrisome because people instinctively associate unhealthy foods with better taste [32]. Interestingly, a study analyzing families’ demand for food prepared away from home determined that price was the most attractive factor for families when choosing a restaurant, followed by taste [33]. One meal prepared away from home adds approximately 134 calories to the daily energy intake [34]. Knowledge of personal energy requirements is a fundamental principle of nutrition literacy, without which controlling one’s energy intake may be challenging. Half of the respondents could calculate their personal energy requirements and such knowledge was found to be correlated with younger age, higher level of education, normal BMI classification based on self-reported height and weight, no chronic disease, and majoring in nutrition or food science. In the United States, it was correlated with being female, white, and having a higher level of education and income but not with any BMI classification [35].

The menu calorie labeling policy enables consumers to make informed food choices. The policy could aid consumers in accurately estimating the calorie content of ordered foods, which are often served in large amounts compared to homemade meals [36]. An analysis of online food ordering application menus around the world found that most food items were energy-dense and had low nutritional content [37–39]. A systematic review and meta-analysis of international evidence found conflicting associations between noticing calorie information on menus and its effects on foods ordered or selected [40]. In our sample, less than half of the respondents were interested in viewing calorie information on online food ordering application menus or had noticed it. Earlier studies in Saudi Arabia found that 50–60% of consumers were not interested in viewing or did not notice calorie information on online food ordering application menus, while the rest were either very interested or somewhat interested [14, 30]. Interest in viewing calorie information on online food ordering application menus was linked to younger age and higher level of education, as in a previous study in Saudi Arabia [30]. In the United States, those who frequently noticed the menu calorie labeling policy in restaurants were more likely to be female, younger, overweight or obese, and to have a higher level of education and income [41]. Notably, males were more likely than females to notice calorie information on online food ordering application menus, which is inconsistent with previous findings in Saudi Arabia and internationally, where females were most likely to consider calories in menus. This could be due to the uneven gender representation in our sample (83.1% females) [41–45]. Research has shown that displaying calorie information on menus significantly reduces the number of calories ordered and consumed, indicating that it serves as an inexpensive and wide-reaching public health policy [40]. Half of the participants responded positively to the displayed calorie information on online food ordering application menus, and they mostly utilized it to monitor their calories intake to maintain body weight and estimate the nutritional content of ordered meals. Previous research conducted in Saudi Arabia found that noticing calorie information altered consumers’ order choice, portion size, and limited fast-food ordering [15, 30, 46]. A population-based study in the United States found that calorie information on menus was mainly used to avoid ordering energy-dense items and to minimize ordered portions [45]. Research on calorie information in menus is lacking because it is often designed as an observational study, from which no causal inferences can be drawn, or conducted in laboratory settings, which may fail to reflect real-world situations [47].

The findings of this study should be interpreted with caution, since the sample may not be representative due to inadequate gender distribution and convenience sampling. The gender imbalance is one limitation of this study. It is essential to understand that the number of female participants outweighed the male participants, which could introduce a possible bias and limit the generalizability of the findings. Studies have reported that females pay more attention to food labeling than men do which could be a potential reason why more females participated in the study [48]. In addition, after rerunning the analysis without male participants, we found that the exclusion of male participants did not significantly change the overall results and the outcomes remained consistent. The patterns observed in the initial analysis were more or less preserved, suggesting that gender may not have a significant effect on the results. Moreover, some participants may not have an accurate perception of the influence of menu labelling on them. Nevertheless, the findings offer a starting point for the public’s response to calorie labeling in online food ordering application menus and demonstrates the need to increase public awareness about calorie labeling on menus to aid in the success and effectiveness of calorie labeling in online food ordering applications as a tool to promote balanced energy intake, and in turn the population’s health.

## Conclusions

In conclusion, the public’s response to noticing and viewing calorie information on online food ordering application menus was varied. Calorie information was mostly used for weight maintenance and estimation of nutritional content when ordering. Dinner was the most frequently ordered meal and fast-food restaurants and coffee shops were the most popular options. Saving time and effort were the chief reasons behind the use of online food ordering applications, whereas taste and cravings were the primary drivers behind the respondents’ restaurant choices. The ability to calculate energy requirements and interest in viewing calorie information on online food ordering application menus were linked to younger age and a higher level of education. These findings highlight the need to increase public awareness about calorie labeling on menus to aid in the success and effectiveness of the calorie labeling in online food ordering application as a tool to promote balanced energy intake.

### Electronic supplementary material

Below is the link to the electronic supplementary material.

Supplementary Material 1: The English version of?The Consumers? Behaviors toward Display the Calorie Information of Meals on Online Food Ordering Applications in Saudi Arabia? Questionnaire

## Data Availability

The data that support the findings of this study are available upon reasonable request. Please contact Ms. Sarah Alkhunein via an Email: Smkhunein@sfda.gov.sa.

## Abbreviations

BMI: Body mass index
COVID-19: Coronavirus disease 2019
SFDA: Saudi Food and Drug Authority
