# Supplementary material for: Response of the consumers to the menu calorie-labeling on online food ordering applications in Saudi Arabia
Source: BMC Nutr. 2024 Feb 2;10:25. doi: 10.1186/s40795-024-00829-x (PMC10836020; doi:10.1186/s40795-024-00829-x)
Supplement: Supplementary file 1 — Supplementary Material 1: The English version of?The Consumers? Behaviors toward Display the Calorie Information of Meals on Online Food Ordering Applications in Saudi Arabia? Questionnaire [file 40795_2024_829_MOESM1_ESM.docx]

**The English version of “The Consumers’ Behaviors toward Display the Calorie Information of Meals on Online Food Ordering Applications in Saudi Arabia” Questionnaire**

**Section 1: Sociodemographic characteristics**

1. **Age (Years) :** ______________
2. **Gender:**

- Male
- Female

1. **Marital status:**

- Single
- Married
- Divorced
- Widowed

1. **Highest educational level**

- Below secondary school
- Secondary school/equivalent
- Bachelor`s degree
- Postgraduate degree

1. **Job status:**

- Student
- Employed
- Unemployed
- Self-employed
- Retired

1. **Field of study:**

- Nutrition or food science
- Medical specialties (Medicine, Pharmacy, Applied medical sciences…etc.)
- Science (Chemistry, Biology…etc.)
- Literary (Languages, Geography…etc.)
- Management (Human resources, General management)
- Non-specific

1. **Monthly income, SR (US dollars);**

- Less than 2000 (<533$ USD)
- 2000-5000 (533-1333$ USD)
- 5001-7000 (1334-1866$ USD)
- 7001-10000 (1867-2666$ USD)
- More than 10000 (>2666$ USD)

1. **Weight (kg):** ___________
2. **Height (cm):** ___________
3. **Have you been diagnosed by the following conditions before*?**

- Cardiovascular diseases and high blood cholesterol
- Cancer
- Diabetes
- Kidney diseases
- Liver diseases
- Gastric ulcer
- Anemia
- Osteoporosis
- No, I haven't been diagnosed with any of the above
- Other

**Section 2:** **Preference of ordering from online food ordering applications**

1. **Do you prefer to order directly from the restaurant or online food ordering applications?**

- Online food ordering applications
- Restaurants

1. **What is the reason behind using online food ordering applications*?**

- The different price and offers compared with ordering directly from the restaurant
- Saving time and effort
- The ease of access to restaurants and shops compared with ordering from a restaurant directly
- The variety of options for restaurants and shops available on the apps

1. **Has your use of online food ordering applications increased during the COVID-19 pandemic?**

- No
- Yes

**Section 3:** **Frequency of ordering from online food ordering applications**

1. **What type of restaurants or shops do you order from?***

- Fast-food restaurants
- International food restaurants
- Traditional food restaurants
- Healthy food restaurants
- Vegetarian restaurants
- Desserts shops
- Beverages shops
- Coffee shops
- Others

1. **What is the reason behind choosing a specific restaurant from the application?***

- Meal price
- Delivery price
- Delivery speed
- Healthiness
- Taste
- Reviews from others
- Pictures displayed on menus
- Craving a specific food
- The desire of others to eat certain food
- Level of hygiene and quality
- Other reasons

1. **What type of meal do you order the most from online food ordering applications?**

- Breakfast
- Lunch
- Dinner
- Snacks
- Beverages

1. **How frequently do you use online food ordering applications?**

- Once per week
- 2-4 times per week
- 5-6 times per week
- Once per day
- 2 or more times per day

1. **What time of the day do you mostly order from online food ordering applications?**

- Morning (6 am - 12 pm)
- Afternoon (1 pm - 6 pm)
- Evening (7 pm - 12 am)
- Post-midnight (1 am - 5 am)
- No specific time

1. **When you order from food delivery apps, you order:**

- For myself
- For myself, and a family member or friend
- For a family member or friend only

**Section 4: Public perceptions and knowledge of calorie information on online food ordering application menus**

1. **Do you know how to calculate your daily calorie requirement?**

- Yes
- No

1. **When you order from an online food ordering application, do you notice any caloric information displayed on the menu?**

- Yes
- No
- Sometimes

1. **Are you interested in viewing calorie information on online food ordering application menus?**

- Yes
- No
- Sometimes

**Section 5: Impact of displaying calorie information of meals on online food ordering applications on public food choices**

1. **Dose displaying calorie information on menus in online food ordering application positively affect your food choices?**

- Yes
- No
- Sometimes

1. **Mostly I use the calorie information*:**

- To not exceed the total calories of the day for the purpose of maintaining weight
- To plan other meals that I will eat later in the day for the purpose of gaining weight
- To plan other meals that I will eat later in the day for the purpose of losing weight
- To estimate the amount of nutrients in a meal (ex: amount of fats, protein, or carbohydrate)
- Other reason

***Participant is allowed to choose more than one option.**
